# Nursing care for patients with endometriosis in Aotearoa New Zealand: a survey study

**DOI:** 10.1177/17449871261430232

**Published:** 2026-04-27

**Authors:** Katherine Ellis, Alina Meador, Anna Ponnampalam, Rachael Wood

**Affiliations:** PhD Student, Department of Chemical and Process Engineering, University of Canterbury, Christchurch, New Zealand; PhD Student, School of Population Health, Faculty of Medical and Health Sciences, University of Auckland, Auckland, New Zealand; Senior Research Fellow, Department of Physiology, Faculty of Medical and Health Sciences, University of Auckland, Auckland, New Zealand; Senior Lecturer, Department of Chemical and Process Engineering, University of Canterbury, Christchurch, New Zealand

**Keywords:** advocacy, chronic pain, endometriosis, nursing, patient management, specialisation

## Abstract

**Background:**

Endometriosis is a chronic condition where patients face challenging care journeys. There is minimal literature regarding perspectives of nurses on their approach to caring for endometriosis patients.

**Aims:**

This paper assesses Aotearoa New Zealand nurses’ awareness and understanding of New Zealand guidelines, perceptions of endometriosis care, perspectives of their role in this care and interest in endometriosis-specialist nursing roles.

**Methods:**

Two hundred and ninety-two nurses completed an online, anonymous, exploratory, descriptive survey about their knowledge, view of the importance of endometriosis nursing roles and personal interest in becoming an endometriosis-specialist nurse.

**Results:**

16.1% of nurses perceived they knew enough about endometriosis for their routine practice. The majority (72.9%) of nurses surveyed indicated they were personally interested in becoming an endometriosis-specialist nurse, with some willing to dedicate several years to becoming qualified for this role.

**Conclusion:**

The results of this study indicate both a self-perceived lack of knowledge amongst nurses regarding endometriosis as a topic, as well as a desire for readily available education and upskilling regarding nursing for patients with the condition. The authors hypothesise that investment into having endometriosis-specialist nurses may alleviate some of the pressure from the primary and secondary health system care of endometriosis patients.

## Introduction

Endometriosis is a chronic condition, characterised by the presence of tissue similar to the lining of the uterus, outside of the uterus (World Health Organization, 2023). The condition is typified for patients by pain, fatigue (Lemaire, 2004), infertility (Bonavina and Taylor, 2022), reduced health-related quality of life (Márki et al., 2017), substantial life disruptions (Hansen et al., 2013) and financial burdens (Simoens et al., 2012). Endometriosis patients in Aotearoa New Zealand have reported challenging journeys through diagnosis and management of endometriosis. Patients have reported that their symptoms are dismissed or trivialised, their diagnostic journeys are incredibly challenging, and the way in which they are treated by their medical practitioners has a substantial capacity to improve their journey or to create barriers (Ellis and Wood, 2025).

Patients in Aotearoa New Zealand on average experience a 9–10-year delay to diagnosis (Ellis and Wood, 2024; Tewhaiti-Smith et al., 2022) and will visit an average of five general practitioners (GPs) prior to diagnosis (Tewhaiti-Smith et al., 2022). In an interview study with nine GPs, it was suggested that one way to improve the care provided to endometriosis patients in Aotearoa New Zealand was to have specialised endometriosis GPs or nurses (Ellis et al., 2025). An international study that assessed the comments of 222 endometriosis patients about medical practitioners indicated practitioners were predominantly viewed as barriers, not facilitators, of endometriosis care (Mikesell and Bontempo, 2023). This was due to a perceived lack of expertise, along with neglect and dismissiveness (Mikesell and Bontempo, 2023). The issue of lacking expertise about endometriosis in the general population of medical practitioners has also been identified as an issue in a sample of 13 endometriosis specialists in Australia (Fallon et al., 2024). When medical practitioners are seen as facilitators to endometriosis care, these relationships can be described as life-changing for patients, supporting the concept that the attitudes and behaviours of medical practitioners are vital in endometriosis patient care experiences (Ellis and Wood, 2025; Mikesell and Bontempo, 2023).

In a Catalan cohort of endometriosis patients, nurses were the second most visited medical practitioners following GPs (Medina-Perucha et al., 2022). Specialist nurse-led multidisciplinary clinics have been proposed as a means to reduce waitlists. These nurses can provide consultations that involve a comprehensive history taking, allow the patient to feel heard, explain endometriosis pain mechanisms and develop realistic goals and the methods to achieve those goals (Cambitzi and Nagaratnam, 2013). These clinics and roles have the capacity to reduce the healthcare burden of chronic pain patients (such as endometriosis patients) from both primary and secondary care sectors. Attendance of fibromyalgia patients to a nurse-led chronic musculoskeletal pain clinic in the UK significantly reduced hospital and GP visits by those patients versus prior to treatment at the clinic, and in comparison to those who did not attend the clinic (Ryan et al., 2012). At this clinic, the nurse consultant confirmed the fibromyalgia diagnosis, reviewed current and past treatment plans, holistically evaluated the patient’s life and then developed an individualised self-management plan (Ryan et al., 2012).

In Aotearoa New Zealand, GPs have highlighted the challenges associated with being too time poor in consultations to be able to provide patients with the in depth holistic care and communication that would benefit the therapeutic relationship (Ellis et al., 2025). Nurse-led primary care services, particularly those incorporating highly trained and specialised nurses, have the capacity to excel in preventative care and chronic disease care (Flaubert et al., 2021; Smolowitz et al., 2015) in a way that is currently missing in the Aotearoa New Zealand context. In particular, nurses are the practitioners who most regularly serve individuals who are less able to access other medical care, or whom are better served with preventative care and management than acute care (Flaubert et al., 2021), a category that fits the chronic nature of endometriosis. Currently practising nurses with a specialisation in endometriosis care have highlighted that under their care patients are presented with a means to share their stories (Cambitzi and Nagaratnam, 2013), and the nurse act as a patient advocate (Norton et al., 2020), enhance the sharing of information and education with the patient (Chen et al., 2018) and limit the exposure of patients to practitioners who do not understand the challenges of the condition (Bach et al., 2016). Although substitution of nurses for primary care doctors in care is not associated with appreciable differences in health outcomes, it has been associated with higher patient satisfaction scores, longer consultations, increased provision of information to patients and a higher number of patients returning for follow-up visits (Laurant et al., 2005, 2018). Patient satisfaction, consultation length, information provision and engagement in follow-up care are all features of endometriosis care that would benefit from improvement in Aotearoa New Zealand (Ellis et al., 2025; Ellis and Wood, 2025).

It may be valuable to develop and utilise a service for endometriosis in Aotearoa New Zealand that is nurse-led, similar to the UK nurse-led chronic musculoskeletal pain clinic (Ryan et al., 2012). This type of service may have the capacity to improve patient satisfaction with the care provided to them through longer consultations, a more holistic approach and improved provision of information about the condition. This service may also have the potential to reduce pressure on other health system services and create the capacity to avoid more costly care needs in the long term (Smolowitz et al., 2015) although this would require further investigation to confirm.

In Aotearoa New Zealand, endometriosis patients have frequent interactions with the health system (Tewhaiti-Smith et al., 2022), but there is limited data available about the perspectives of nurses internationally, and no data in Aotearoa New Zealand regarding their approach to endometriosis patient care. In Aotearoa New Zealand, guidance for endometriosis is predominantly from a non-clinical guideline published in 2020 (Ministry of Health, 2020). This brief guideline includes key signs of endometriosis, the means of investigating endometriosis, management of endometriosis in primary healthcare through non-steroidal anti-inflammatory drugs and hormonal treatment and non-pharmacological pain management (e.g. pain psychology) and the surgical management of patients when referred to secondary and tertiary gynaecological care (Ministry of Health, 2020). These guidelines specify progesterone-only pills (POPs) are the first-line hormonal treatment for patients with presumed or proven endometriosis (Ministry of Health, 2020).

At present, there is no literature or data regarding the role of nurses in endometriosis care in Aotearoa New Zealand. The purpose of this study is to begin the process of closing this key research gap by assessing features of endometriosis care knowledge and practice amongst nurses in Aotearoa New Zealand. This was done by assessing nurse awareness and understanding of the Diagnosis and Management of Endometriosis in New Zealand guidelines, their knowledge, understanding and perceptions of elements of endometriosis care, their perspectives on their roles in endometriosis patient care and the interest in endometriosis-specialist nurse roles in Aotearoa New Zealand. These features were assessed according to the age, guidelines exposure (awareness and having read the New Zealand endometriosis guidelines) and the regularity with which the participating nurses were exposed to gynaecology settings (such as working in an obstetrics and/or gynaecology clinic), training (endometriosis-specific continuing medical education) and consults (frequent or infrequent gynaecology consults).

## Methodology

### Survey design

The survey was adapted from a 2023 to 2024 study of GPs in Aotearoa New Zealand (Ellis et al., 2024). This study used the same questions regarding frequency of consultations, gynaecology training, sufficient knowledge of endometriosis (yes/somewhat/no), awareness and usefulness of national endometriosis guidelines (binary yes/no), first-line treatments for endometriosis as well as alternative management strategies (binary yes/no) and symptoms suggestive for endometriosis (always/sometimes/rarely/never have diagnostic value) as in the published GP questionnaire (Ellis et al., 2024). Regarding the *Diagnosis and Management of Endometriosis in New Zealand* guidelines, nurses were given an open-text question of any comments they had about the guidelines. Nurses were additionally asked to rank eight potential nursing roles for endometriosis for their perception of each role’s importance (providing information about: endometriosis as a disease, endometriosis diagnosis, endometriosis-related fertility implications, endometriosis treatments; advocating for endometriosis patients; providing emotional support to endometriosis patients; discussing potential lifestyle changes [e.g. diet or exercise changes]; and referring endometriosis patients to local or online support groups). These roles were selected from a review of literature to discern appropriate roles to assess (Chen et al., 2018; Norton et al., 2020; Wedell et al., 1985). Finally, the participating nurses rated whether they would personally be interested in becoming an endometriosis-specialist nurse (7-point Likert scale from very disinterested to very interested), and if so, how long they would be willing to dedicate to training for this role (open-text).

To establish the validity of content to the practice of nurses in Aotearoa New Zealand, the questionnaire was iteratively reviewed and assessed by nurses from a range of practice settings through one-on-one discussions, and a final group panel of nurses to assess the final questionnaire (full questionnaire in supplemental material). This questionnaire has not yet gone through a validation process since the study is novel and exploratory in a research area with minimal existing data internationally and no validated tools to date.

### Recruitment

To recruit nurses, an invitation with the survey link and information sheet was emailed to the managers of practices where nurses may come into contact with endometriosis patients. In total, 954 practices were contacted by email using publicly available contact details through clinic webpages and HealthPages (an online health services directory). Of the contacted practices, 869 were primary care clinics, 36 women’s health or obstetrics and gynaecology clinics, 28 Māori health-related services and 21 after-hours or hospital services. Power calculations were not made prior to recruitment due to the exploratory nature of the study, and the lack of prior literature in the area frown which to draw intended effect sizes. The survey was estimated in the invite to take 5–10 minutes and was kept short in an attempt to maximise the sample size. There was no reimbursement for participation. During the period of recruitment (September 2024 to May 2025), a maximum of two follow-up emails to clinics were sent. It is impossible to calculate the response rate to the invites due to the nature of the recruitment method being via clinic managers rather than to nurses directly. All nurses who were over the age of 18, practising in Aotearoa New Zealand and consented to participate in the survey were included. No further exclusion criteria were applied.

### Data analysis

All statistical analyses were conducted using Qualtrics StatsIQ at a confidence level of 95% (⍺ = 0.05). Comparisons between two categorical groups (more and less experienced nurses, those with and without endometriosis continuing medical education, frequent and infrequent gynaecological consults, those who were and were not aware of the guidelines, those who had and had not read the guidelines, those who did or did not work in a specialised obstetrics and/or gynaecology workplace and ratings of roles as high or low priority) were done with Fisher’s exact test. Comparisons between more than two categorical groups (age groups, and perception of personal endometriosis knowledge [as ‘yes’ sufficient, ‘somewhat’ or ‘no’ not sufficient]) were done with Pearson’s χ^2^ test. Interest in becoming an endometriosis-specialist nurse was measured on a 7-point Likert scale from ‘very interested’ to ‘very disinterested’. The data were transformed by grouping ‘very interested’, ‘interested’ and ‘somewhat interested’ into ‘interested’ and ‘very disinterested’, ‘disinterested’, ‘somewhat disinterested’ and ‘neutral’ into ‘not interested’ for a non-ordered nominal metric which avoided small sub-group sample sizes for statistical analysis. Non-dichotomised results are available in supplemental materials. Comparisons involving a continuous variable, such as the rank of a roles value from 1 to 8, were performed with *t*-tests. The null hypothesis in all cases was that there were no differences between the groups. Open-text answers were thematically coded in an inductive, semantic manner (Willig and Rogers, 2017) according to the explicit meanings of the answers, which were grouped into distinct final sentiments, as was done in a comparable GP survey (Ellis et al., 2024).

### Ethics

The approach of this exploratory, descriptive, online, anonymous study was reviewed and approved by the University of Canterbury Human Research Ethics Committee (Ref: 2024/107/LR-PS) as well as the Ngāi Tahu Consultation and Engagement Group for inclusion of Māori participants and collection of their data. At the onset of the survey, participants were provided with the information sheet to read. Once participants reached the bottom of the information sheet, to proceed they agreed that submitting their responses was consent to participate. All data were anonymous, and no identifying information was captured or stored with survey answers. Nurses who signed up to participate in a simultaneously recruited for semi-structured interview study were directed to a separate survey to keep their contact details separate from their survey answers. This study was reported according to STROBE guidelines (supplementary material; von Elm et al., 2007).

## Results

### Participant cohort

Of the 304 nurses who began the survey, 292 completed at least 75% of the survey and had their answers included in the final dataset (Table 1). The sample was diverse with regard to age, ethnicity, region and rurality of practice, location of training, type of nursing role and workplace, speciality of workplace, gynaecology and endometriosis-specific training completion and gynaecology consult frequency (Table 1). The nurses were primarily female, had done their nursing training in Aotearoa New Zealand and had not done further formal gynaecology training or endometriosis-specific continuing medical education (CME). The 222 nurses from primary care clinics represent 5.9% of primary healthcare nurses in Aotearoa New Zealand(Nursing Council of New Zealand; Te Kaunihera Tapuhi o Aotearoa, 2024).

**Table 1.** Nurse survey participant cohort.

Variable	*N*	%
Total	292	100.0
Gender		
Female	286	97.9
Male	6	2.1
Age (years)		
20–29	54	18.5
30–39	77	26.4
40–49	54	18.5
50–59	61	20.9
60+	46	15.8
Ethnicity		
Pāhekā/New Zealand European	222	76.0
New Zealand Māori	30	10.3
Asian	18	6.2
Pasifika	12	4.1
British or European	23	7.9
Australian	3	1.0
African	2	0.7
Middle Eastern	1	0.3
North American	3	1.0
Latin American	1	0.3
Region		
Auckland/Tāmaki Makaurau	67	22.9
Canterbury/Waitaha	55	18.8
Wellington/Te Whanga-nui-a-Tara	37	12.7
Otago/Ōtākou	26	8.9
Manawatū-Whanganui	16	5.5
Southland/Murihiku	17	5.8
Northland/Te Tai Tokerau	12	4.1
Waikato	18	6.2
Hawke’s Bay/Te Matau-a-Māui	13	4.5
Bay of Plenty/Te Moana-a-Toi	11	3.8
Marlborough/Te Tau Ihu-o-te-Waka	8	2.7
Nelson/Whakatū	7	2.4
Taranaki	3	1.0
Gisborne/Te Tairāwhiti	2	0.7
Tasman/Te Tai-o-Aorere	2	0.7
West Coast/Te Tai Poutini	1	0.3
Location of training		
New Zealand	265	90.8
UK or Europe	25	8.6
Australia	15	5.1
Asia	3	1.0
North America	3	1.0
South America	1	0.3
Rurality		
Rural	29	9.9
Semi-rural	44	15.1
Urban	219	75.0
Nursing role		
Registered Nurse	216	74.0
Enrolled Nurse	5	1.7
Nurse Practitioner	11	3.8
Senior Nurse	26	8.9
Clinical Nurse Specialist	8	2.7
Nurse Co-ordinator	8	2.7
Nurse Prescriber	12	4.1
Nurse Manager	5	1.7
Kaimanaaki^a^	1	0.3
Workplace		
Primary care clinic	244	83.6
After-hours/Emergency	7	2.4
Hospital	10	3.4
Community/NGO	18	6.2
Private Clinics	9	3.1
Medicinal Cannabis Clinic	1	0.3
Māori Health Clinic	1	0.3
Sexual Assault Assessment and Treatment Service	1	0.3
Telehealth Clinic	1	0.3
OBGYN speciality workplace		
No	226	77.4
Yes – obstetrics only	2	0.7
Yes – obstetrics and gynaecology	19	6.5
Yes – gynaecology only	45	15.4
Gynaecology qualifications		
No	271	92.8
Yes – internship	2	0.7
Yes – post-graduate	17	5.8
Yes – diploma	2	0.7
Endometriosis-specific CME		
Yes	30	10.3
No	262	89.7
Gynaecology consult frequency		
Every day	49	16.8
Several per week	87	29.8
Several per month	115	39.4
Less than once per month	30	10.3
Never	11	3.8

a Te Reo Māori term for a designated supporter.

### The Diagnosis and Management of Endometriosis in New Zealand guidelines

Amongst the cohort of 292 nurses, 31.5% were aware of the guidelines, whereas only 13.0% had read them. Amongst the 38 nurses who had read the guidelines, only one felt they were not useful to their practice. Nurses who were nurse practitioners, senior nurses, clinical nurse specialists (CNSs) or nurse prescribers were more likely than enrolled or registered nurses to be aware of the guidelines (*p* = 0.0107) and to have read them (*p* = 0.0245; Table 2). Those who had done endometriosis-specific CME were more likely to be aware (*p* < 0.00001) and to have read the guidelines (*p* = 0.000401) than those who had not. Nurses with several gynaecology consults per week or more were also more likely to have read the guidelines versus those who had several gynaecology consults per month or fewer (*p* = 0.0141).

**Table 2.** Differences in groups regarding awareness of the guidelines and having read them^a^ and for their perception of if their knowledge about endometriosis is sufficient for their routine practice.^b^

Comparison Groups	*N*	Aware	Not aware		*p*
ENs and RNs	221	27.6%	72.4%		0.0107
NPs, SNs and CNSs	57	45.6%	54.4%		
Endo CME+	30	70.0%	30.0%		<0.00001
Endo CME−	262	27.1%	72.9%		
	*N*	Read	Not Read		
ENs and RNs	221	10.4%	89.6%		0.0245
NPs, SNs and CNSs	57	22.8%	77.2%		
Endo CME+	30	36.7%	63.3%		0.000401
Endo CME−	262	10.3%	89.7%		
Frequent consults	136	18.4%	81.6%		0.0141
Infrequent consults	156	8.3%	91.7%		
	*N*	Yes	Somewhat	No	*p*
Aware	92	30.4%	52.2%	17.4%	<0.00001
Not aware	200	9.5%	41.5%	49.0%	
Read	38	39.5%	52.6%	7.9%	<0.00001
Not read	254	12.6%	43.7%	43.7%	
Specialised	66	22.7%	51.5%	25.8%	0.0298
Not specialised	226	14.2%	42.9%	42.9%	
Endo CME+	30	36.7%	50.0%	13.3%	0.000660
Endo CME−	262	13.7%	44.3%	42.0%	

a ‘ENs and RNs’ (enrolled nurses and registered nurses combined), ‘NPs, SNs and CNSs’ (nurse practitioners, nurse prescribers, senior nurses and clinical nurse specialists combined), ‘Endo CME +’ (endometriosis-specific continuing medical education completed), ‘Endo CME-’ (endometriosis-specific continuing medical education not completed), ‘Frequent’ (several per week or more gynaecology consults), ‘Infrequent’ (several per month or less gynaecology consults), ‘Aware’ (were aware of the *Diagnosis and Management of Endometriosis in New Zealand* guidelines), ‘Not Aware’ (not aware of the guidelines), ‘Read’ (had read the guidelines) and ‘Not Read’ (had not read the guidelines).

b ‘Specialised’ (workplace had a gynaecology and/or obstetrics specialisation), ‘Not Specialised’ (workplace did not have this kind of specialisation), ‘Endo CME +’ (endometriosis-specific continuing medical education completed) and ‘Endo CME-’ (endometriosis-specific continuing medical education not completed). ‘Yes’, ‘Somewhat’ and ‘No’ are the answers of nurses to the question: ‘Do you feel you know enough about endometriosis for your routine practice?’.

When given the opportunity to comment on the *Diagnosis and Management of Endometriosis in New Zealand* guidelines, there were three key themes: the guidelines were under-employed, the guidelines were generally useful and that more support was needed to facilitate utilisation of the guidelines. The comments made more than once were: that respondents should or would now read the guidelines (*N* = 6); respondents had read portions of the guidelines (*N* = 4) or had not read them at all (*N* = 2); the guidelines may need to be updated (*N* = 2); the existence of the guidelines needs more awareness amongst nurses (*N* = 2); the guidelines were somewhat helpful or a good summary (*N* = 3) and that respondents wanted more educational opportunities regarding endometriosis (*N* = 2). It was highlighted by one nurse that more was needed than just the 2020 guidelines, ‘Training sessions would be of great value to this practice in general’ (Nurse 285).

### Knowledge regarding endometriosis for routine practice

Regarding personal perception of their understanding of endometriosis and whether they felt it was enough for their routine practice, only 16.1% felt that ‘yes’ it was sufficient. The nurses more likely to view they had sufficient knowledge (Table 2) were those with awareness of the guidelines (χ^2^(2, *N* = 292) = 34.9, *p* < 0.00001), who had read the guidelines (χ^2^(2, *N* = 292) = 26.3, *p* < 0.00001) or had conducted endometriosis-specific CME (χ^2^(2, *N* = 292) = 14.6, *p* = 0.000660). Nurses who had a workplace with an obstetrics and/or gynaecology specialisation were less likely to have the sense that they lacked sufficient knowledge (χ^2^(2, *N* = 292) = 7.02, *p* = 0.0298).

### Rating of diagnostic value of endometriosis symptoms

**Figure 1.**
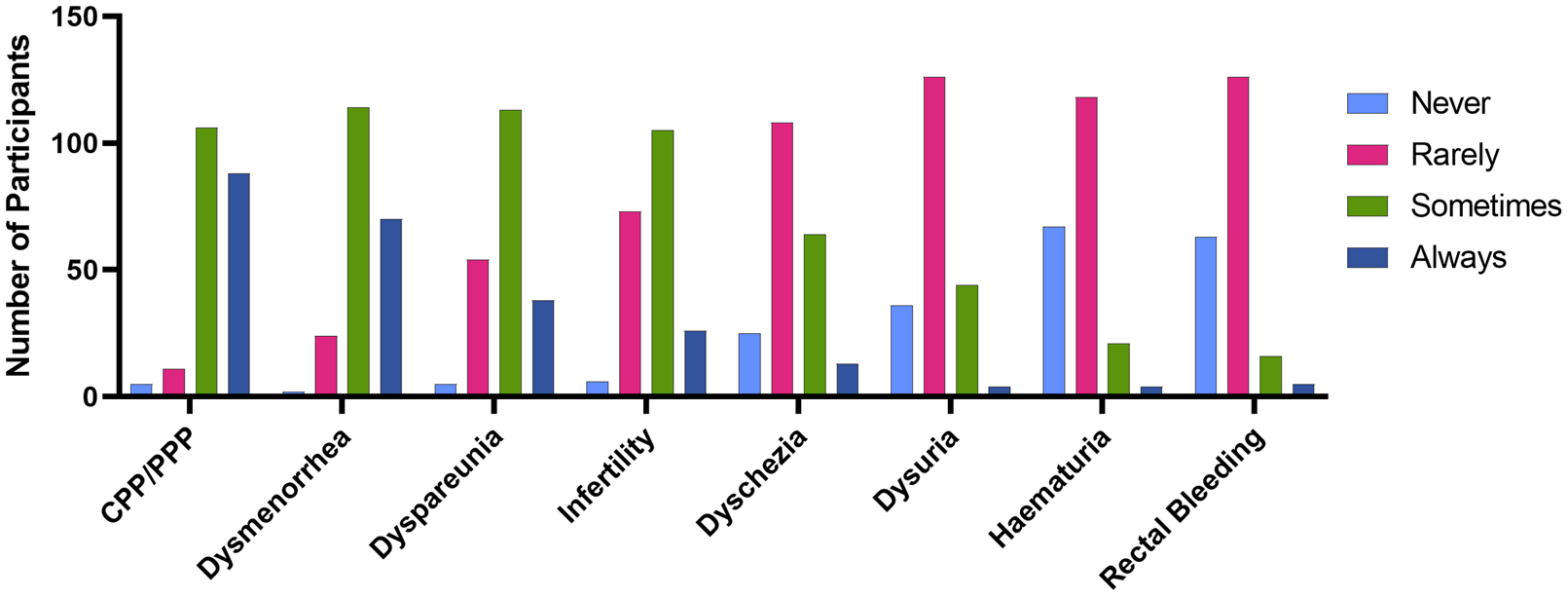
View of nurses (*N* = 210) on the frequency of diagnostic value of the endometriosis symptoms of chronic or persistent pelvic pain (CPP/PPP), dysmenorrhea, dyspareunia, infertility, dyschezia, dysuria, haematuria and rectal bleeding. Participants rated the symptoms on a scale of 1–4, with 1 representing the symptom ‘Never’, 2 ‘Rarely’, 3 ‘Sometimes’ and 4 ‘Always’ having diagnostic value for endometriosis.

The nurses in this study rated chronic/persistent pelvic pain (PPP) for a period over 6 months, dysmenorrhea (painful periods), deep dyspareunia (painful sex), infertility, dyschezia (painful defecation), dysuria (painful urination), haematuria (bleeding with urination) and painful rectal bleeding for the diagnostic value they viewed them to have for endometriosis (Figure 1). These symptoms were given a numerical value by the nurses depending on whether they viewed that symptom to never (1), rarely (2), sometimes (3) or always (4) have diagnostic value for endometriosis. The nurses ranked from most to least diagnostic value for endometriosis (average ± standard deviation, median [inter-quartile range]):

1. PPP (3.32 ± 0.68, 3 [3, 4]),
2. Dysmenorrhea (3.20 ± 0.67, 3 [3, 4]),
3. Deep dyspareunia (2.88 ± 0.72, 3 [2, 3]),
4. Infertility (2.72 ± 0.71, 3 [2, 3]),
5. Dyschezia (2.31 ± 0.76, 2 [2, 3]),
6. Dysuria (2.08 ± 0.67, 2 [2, 2]),
7. Haematuria (1.82 ± 0.68, 2 [1, 2]),
8. Painful rectal bleeding (1.82 ± 0.67, 2 [1, 2]).

The median ratings indicated the nurses of this study viewed PPP, dysmenorrhea, deep dyspareunia and infertility as ‘sometimes’ having diagnostic value, and the remaining symptoms ‘rarely’ having diagnostic value. The only significant differences in ratings between groups were for deep dyspareunia and haematuria. For deep dyspareunia, nurse practitioners, senior nurses, CNSs and nurse prescribers rated deep dyspareunia as having higher diagnostic value (mean 3.06/4.00 (*N* = 47) vs. 2.82/4.00 (*N* = 155), *t*-test, *p* = 0.0485) than enrolled and registered nurses, and haematuria as having lower diagnostic value (average 1.55/4.00 (*N* = 47) vs. 1.89/4.00 (*N* = 155), *t*-test, *p* = 0.00129). Those whose workplace had an obstetrics and/or gynaecology specialisation also rated haematuria as having lower diagnostic value than those without (average 1.63 (*N* = 46) vs. 1.87 (*N* = 164), *t*-test, *p* = 0.0136).

### Recommended treatment and management

**Figure 2.**
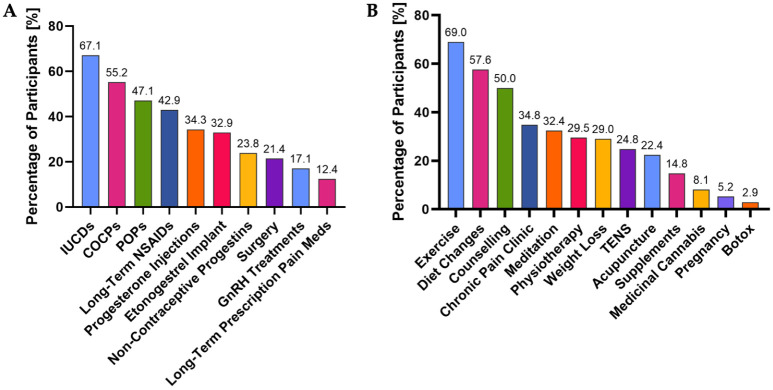
View of nurses (*N* = 210) on (a) the treatments they consider to be first-line for those presenting with endometriosis symptoms, and (b) the alternative management strategies they recommend to those presenting with endometriosis symptoms. IUCDs: intrauterine contraceptive devices; COCPs: combined oral contraceptive pills; POPs: progesterone-only pills; NSAIDs: non-steroidal anti-inflammatory drugs; GnRH: gonadotrophin-releasing hormone; TENS: transcutaneous electrical nerve stimulation.

When asked about what they viewed as the first-line treatments for symptomatic endometriosis, the only treatments selected by a majority were intrauterine contraceptive devices (IUCDs) at 67.1% and combined oral contraceptive pills (COCPs) at 55.2% (Figure 2A). With regard to alternative methods (Figure 2B), they recommended to patients presenting with endometriosis symptoms, a majority recommended exercise (69.0%) and diet changes (57.6%), whereas 50.0% also recommended counselling/talk-based therapy. There were differences in recommendations based upon age, self-perception of knowledge about endometriosis, whether the nurse had conducted endometriosis-specific CME, type of nursing role, awareness of the guidelines and having read the guidelines (Table 3).

**Table 3.** Differences in the recommendations of nurses of first-line treatments and alternative management strategies based upon age, perception of knowledge, training and upon awareness of, and having read, the *Diagnosis and Management of Endometriosis in New Zealand*. ‘Aware’ (were aware of the *Diagnosis and Management of Endometriosis in New Zealand* guidelines), ‘Not Aware’ (not aware of the guidelines), ‘Read’ (had read the guidelines) and ‘Not Read’ (had not read the guidelines).^a^

Age (years)	20–29	30–39	40–49	50–59	60+	*p*	DF	χ2
*N*	38	57	40	44	31			
NSAIDs +	57.9%	22.8%	40.0%	43.2%	64.5%	< 0.001	4	18.9
NSAIDs −	42.1%	77.2%	60.0%	56.8%	35.5%			
Weight loss +	15.8%	29.8%	47.5%	27.3%	22.6%	0.0320	4	10.6
Weight loss −	84.2%	70.2%	52.5%	72.7%	77.4%			
Chronic pain clinic +	65.8%	26.3%	22.5%	43.2%	16.1%	< 0.001	4	26.7
Chronic pain clinic −	34.2%	73.7%	77.5%	56.8%	83.9%			
TENS +	47.4%	24.6%	20.0%	20.5%	9.7%	0.004	4	15.1
TENS −	52.6%	75.4%	80.0%	79.5%	90.3%			
Knowledge	Yes		Somewhat	No		*p*	DF	χ^2^
*N*	38		90	82				
POPs +	71.1%		44.4%	39.0%		0.004	2	11.1
POPs −	28.9%		55.6%	61.0%				
COCPs +	73.7%		47.8%	54.9%		0.027	2	7.3
COCPs −	26.3%		52.2%	45.1%				
Non-cont progestins +	44.7%		23.3%	14.6%		0.002	2	13.0
Non-cont progestins −	55.3%		76.7%	85.4%				
IUCDs +	84.2%		63.3%	63.4%		0.047	2	6.1
IUCDs −	15.8%		36.6%	36.6%				
Chronic pain clinic +	28.9%		45.6%	25.6%		0.016	2	8.2
Chronic pain clinic −	71.1%		54.4%	74.4%				
Counselling +	73.7%		45.6%	43.9%		0.005	2	10.5
Counselling −	26.3%		54.4%	56.1%				
Diet changes +	78.9%		57.8%	47.6%		0.005	2	10.5
Diet changes −	21.1%		42.2%	52.4%				
Physiotherapy +	47.4%		31.1%	19.5%		0.007	2	9.9
Physiotherapy −	52.6%		68.9%	80.5%				
CME	CME+			CME-		*p*		
*N*	26			184				
POPs +	69.2%			44.0%		0.020		
POPs −	30.8%			56.0%				
Codeine +	26.9%			10.3%		0.025		
Codeine −	73.1%			89.7%				
IUCDs +	88.5%			64.1%		0.014		
IUCDs −	11.5%			35.9%				
Progesterone injections +	53.8%			31.5%		0.029		
Progesterone injections −	46.2%			68.5%				
Physiotherapy +	57.7%			25.5%		0.002		
Physiotherapy −	42.3%			74.5%				
Role	ENs and RNs			NPs, SNs and CNSs		*p*		
*N*	155			47				
Progesterone injections +	27.1%			59.6%		< 0.001		
Progesterone injections −	72.9%			40.4%				
Pregnancy +	3.2%			12.8%		0.021		
Pregnancy −	96.8%			87.2%				
Botox +	1.3%			8.5%		0.027		
Botox −	98.7%			91.5%				
Weight loss +	25.8%			42.6%		0.044		
Weight loss −	74.2%			57.4%				
Awareness	Aware			Not Aware		*p*		
*N*	67			143				
Chronic pain clinic +	46.3%			29.4%		0.020		
Chronic pain clinic −	53.7%			70.6%				
Exercise +	82.1%			62.9%		0.006		
Exercise −	17.9%			37.1%				
Counselling +	71.6%			39.9%		< 0.001		
Counselling −	28.4%			60.1%				
Diet changes +	71.6%			51.0%		0.007		
Diet changes −	28.4%			49.0%				
Physiotherapy +	44.8%			22.4%		0.001		
Physiotherapy −	55.2%			77.6%				
Meditation +	49.3%			24.5%		< 0.001		
Meditation −	50.7%			75.5%				
Read	Read			Not Read		*p*		
*N*	29			181				
Exercise +	89.7%			65.7%		0.009		
Exercise −	10.3%			34.3%				
Acupuncture +	48.3%			18.2%		0.001		
Acupuncture −	51.7%			81.8%				
Counselling +	75.9%			45.9%		0.004		
Counselling −	24.1%			54.1%				
Diet changes +	75.9%			54.7%		0.042		
Diet changes −	24.1%			45.3%				
Physiotherapy +	62.1%			24.3%		<0.001		
Physiotherapy −	37.9%			75.7%				
Meditation +	65.5%			27.1%		<0.001		
Meditation −	34.5%			72.9%				

a NSAIDs: non-steroidal anti-inflammatory drugs, TENS: transcutaneous electrical nerve stimulation, POPs: progesterone-only oral contraceptive pills, COCPs: combined oral contraceptive pills, non-cont progestins: non-contraceptive progestins), IUCDs: intrauterine contraceptive devices. **‘**+**’** means it was selected by respondents, ‘−’ means it was not selected.

Nurses who were 20–29 years or over 60 were more likely than those in other age groups to recommend NSAIDs (*p* = 0.000808), those 40–49 years were more likely to recommend weight loss (*p* = 0.0320), whereas those who were 20–29 years were more likely to recommend chronic pain clinics (*p* = 0.0000229) and Transcutaneous Electrical Nerve Stimulation (TENS) machines (*p* = 0.00443) (Table 3). Those who reported that ‘yes’, they felt they knew enough about endometriosis for their routine practice were more likely to view POPs (*p* = 0.00379), COCPs (*p* = 0.0265), non-contraceptive progestins (*p* = 0.00151) and IUCDs (*p* = 0.0467) as first-line, as well as more likely to recommend counselling (*p* = 0.00536), diet changes (*p* = 0.00531) and physiotherapy (*p* = 0.00717) (Table 3). Meanwhile, those who felt they ‘somewhat’ knew enough were more likely than those with a higher or lower perception of their endometriosis to recommend chronic pain clinics (*p* = 0.0164) (Table 3). Those who had completed endometriosis-specific CME were more likely to recommend POPs (*p* = 0.0204), IUCDs (*p* = 0.0136), progesterone injections (0.0291) and physiotherapy (0.00207); and long-term treatment with prescription-only pain relief (0.0253) as first-line (Table 3). Nurses who were enrolled or registered nurses were less likely to recommend progesterone injections (*p* = 0.0000877), pregnancy (*p* = 0.0211), Botox (*p* = 0.0272) and weight loss (*p* = 0.0439) than nurse practitioners, senior nurses and clinical nurse specialists (Table 3).

With regard to the guidelines, those who were aware of the guidelines were more likely to recommend chronic pain clinics (*p* = 0.0199), exercise (*p* = 0.00622), counselling (*p* = 0.0000278), diet changes (*p* = 0.00678), physiotherapy (*p* = 0.00120) and meditation (*p* = 0.000486) than those who were not aware of the guidelines (Table 3). Those who had read the guidelines were significantly more likely to recommend exercise (*p* = 0.00899), acupuncture (*p* = 0.00114), counselling (*p* = 0.00444), diet changes (*p* = 0.0421), physiotherapy (*p* = 0.000103) and meditation (*p* = 0.0000864) (Table 3). The guidelines encourage non-pharmacological pain management and name exercise, diet, sleep, TENS pain psychology and specialist women’s health physiotherapy as options (Ministry of Health, 2020).

### The roles of nurses in caring for endometriosis patients

Nurses were asked to rank eight roles from most important (1) to least important (8) in caring for endometriosis patients. The nurses ranked from most to least important (average ± standard deviation, median [inter-quartile range]):

1. Providing information about endometriosis as a disease (2.45 ± 1.73, 2 [1, 3]),
2. Providing information about endometriosis diagnosis (3.38 ± 1.84, 3 [2, 5]),
3. Advocating for endometriosis patients (3.99 ± 2.38, 4 [2, 6]),
4. Providing emotional support to endometriosis patients (4.02 ± 1.90, 4 [2, 5]),
5. Providing information about endometriosis treatments (4.22 ± 1.71, 4 [3, 5]),
6. Discussing potential lifestyle changes (e.g. diet or exercise changes) (5.39 ± 1.99, 6 [4, 7]),
7. Providing information about endometriosis-related fertility implications (5.92 ± 1.68, 6 [5, 7]),
8. Referring endometriosis patients to local or online support groups (6.63 ± 1.84, 8 [6, 8]).

The only role to have a bimodal distribution in ranking was advocating for endometriosis patients, with 52 nurses ranking it as the top priority, and 52 nurses ranking it sixth. When the nurses who rated advocacy for patients as 1st to 4th (‘High priority’) were compared with the nurses who rated advocacy as 5th to 8th (‘Low priority’), the only significant difference between the groups was with regard to age (Table 4). Of the 38 nurses aged 20–29 years, 76.3% were in the high priority group, whereas only 25.8% of the 31 nurses aged 60+ were, a significant difference (χ^2^(4, *N* = 210) = 19.1, *p* = 0.000744).

**Table 4.** Comparisons in ranking of roles in caring for endometriosis patients according to age, and in interest in becoming an endometriosis-specialist nurse. Consult frequency refers to gynaecology consults.

Age (years)	20–29	30–39	40–49	50–59	60+	*p*
*N*	38	57	40	44	31	
High priority	76.3%	57.9%	55.0%	45.5%	25.8%	0.000744
Low priority	23.7%	42.1%	45.0%	54.5%	74.2%	
*N*	30	38	24	27	25	
Advocacy as #1	60.0%	41.0%	33.3%	29.6%	8.0%	0.00192
Disease information as #1	40.0%	59.0%	66.7%	70.4%	92.0%	
Interest in Endometriosis-Specialist Nursing	*N*	Interested	Neutral	Disinterested		*p*
Gynaecology consult frequency						
Frequent	100	85.0%	9.0%	6.0%		0.000811
Infrequent	110	61.8%	22.7%	15.5%		
Age (years)						
20–29	38	81.6%	5.3%	13.2%		0.000046
30–39	57	86.0%	8.8%	5.3%		
40–49	40	80.0%	17.5%	2.5%		
50–59	44	68.2%	18.2%	13.6%		
60+	31	35.5%	38.7%	25.8%		

The trend was similar when the group that classed advocating for patients as their number one role (*N* = 52) were compared with the group that classed providing information about endometriosis as a disease as their number one role (*N* = 93). Of the advocacy first group, 34.6% were aged 20–29 years, whereas only 3.8% were aged 60+. Conversely, 12.9% of the disease information group were aged 20–29 years, whereas 24.7% were aged 60+, a significant difference (χ^2^ (4, *N* = 145) = 17.0, *p* = 0.00192).

### Endometriosis-specialist nursing roles

When asked how interested they would personally be in becoming an endometriosis-specialist nurse, 72.9% indicated they were interested, 16.2% were neutral and 11.0% were disinterested. As only 23 nurses expressed they were somewhat to very disinterested in becoming an endometriosis-specialist nurse, those who were interested or not interested were compared as dichotomised variables to avoid the very small samples sizes in sub-groups skewing the results. Those with frequent gynaecology consults were more likely to be interested (χ^2^ (2, *N* = 210) = 14.2, *p* = 0.000811). Meanwhile, those aged 60+ were more likely to be disinterested (χ^2^(8, *N* = 210) = 33.7, *p* = 0.0000462), and those aged 30–39 years were more likely to be interested compared to other age groups (Table 4). These trends were consistent and retained statistical significance when data were non-dichotomised and assessed by one-way ANOVA (supplemental materials). When those who were interested in being an endometriosis-specialist nurse (*N* = 153) were compared with those who were neutral or disinterested (*N* = 57), those who were interested ranked the importance of advocating for patients significantly higher (average 3.78/8 vs. 4.56/8, *t*-test *p* = 0.0263).

When those who were somewhat to very interested in becoming an endometriosis-specialist nurse (*N* = 153) were asked about the maximum amount of time they would personally dedicate to training for this role, there were a range of answers. For those who shared a set period of time, the timing ranged from a couple of hours to 2–3 years. The most common answers of set periods were 12 months (*N* = 18), 6–12 months (*N* = 5), 6 months (*N* = 4) or between 6 and 12 months in a part-time capacity (*N* = 7). Nurses also indicated this training could be on a per week basis (*N* = 31) with the most common per week suggestion being 8 hours per week (*N* = 6). There were also nine nurses who indicated they would be willing to dedicate to this training for ‘as long as it takes’. Emblematic of the depth of the interest in the endeavour for some of the survey participants one nurse shared: ‘Whatever time it takes, it is my dream’ (Nurse 124).

## Discussion

This is the first study to assess the perspectives of nurses in Aotearoa New Zealand on their approach to endometriosis care, an area where there were previously no literature or data. The findings of this study reveal before unidentified gaps in knowledge amongst nurses in Aotearoa New Zealand, an under-utilisation of the inaugural 2020 *Diagnosis and Management of Endometriosis in New Zealand* guidelines, and the prioritisation of specific roles in the care of endometriosis patients, particularly regarding patient education and advocacy. This is also the first study to address the interest of nurses in endometriosis-specialist nursing roles and highlighted that there is a cohort of nurses in Aotearoa New Zealand that are motivated to take on the training that would be required for such a role.

### Nurse perspectives on their knowledge

The nurses within this study indicated that despite there being a substantial interest in the topic of endometriosis, there was a low perception of their endometriosis knowledge. This finding of low knowledge perception is concordant with a Saudi Arabian study of nurses that identified 61% of the 215 participating nurses had low knowledge of endometriosis and only 6% had scores indicative of high knowledge (Elsharkawy et al., 2025). Those with further nursing training (such as being a nurse practitioner or CNS) and those who had done endometriosis-specific CME were more likely to be aware of and to have read the guidelines, but only those who had done endometriosis CME were more likely to perceive they knew enough for their routine practice. Awareness of and having read the guidelines were both associated with nurses perceiving their endometriosis knowledge as sufficient for their routine practice, a trend consistent with a survey of 185 Aotearoa New Zealand GPs (Ellis et al., 2024).

Endometriosis has a diverse symptom profile, which has resulted in symptom profiles alone to date not being considered a sufficiently effective diagnostic tool. The European Society of Human Reproduction and Embryology (ESHRE) guidelines are some of the most comprehensive in the world and include dysmenorrhea (painful periods), dyspareunia (painful sex), dyschezia (painful defecation), dysuria (painful urination), haematuria (bleeding with urination), painful rectal bleeding, particularly when catamenial (occurring with the menstrual cycle) and unexplained infertility, as symptoms individually requiring consideration of endometriosis in the differential (Becker et al., 2022). Neither of these symptoms when assessed in this study were characterised by these nurses as ‘always’ having diagnostic value, nor were they characterised as such by the 185 Aotearoa New Zealand GPs (Ellis et al., 2024).

This low perception of their knowledge about endometriosis may translate overall into the low recognition of first-line treatments amongst the nurses of this cohort. The majority did not identify progesterone injections or POPs as first-line, whereas the majority of Aotearoa New Zealand GPs did (Ellis et al., 2024), and all four are listed in the *Diagnosis and Management of Endometriosis in New Zealand* (Ministry of Health, 2020). Conversely, only 21.4% viewed surgery as first-line (vs. 40.0% of GPs), and 17.1% for GnRH treatments (vs. 24.3% of GPs), which are dissuaded and second-line in the *Diagnosis and Management of Endometriosis in New Zealand*, respectively (Ministry of Health, 2020). Compared with the Aotearoa New Zealand GPs surveys, the nurses were equally likely to recommend exercise, and more likely to recommend counselling, diet changes, chronic pain clinic, weight loss, TENS, acupuncture, supplements, meditation and Botox, and less likely to recommend physiotherapy, and pregnancy (Ellis et al., 2024). Interestingly, awareness of or having read the endometriosis guidelines only significantly influenced the alternative management methods recommended by nurses in this study, not first-line treatments.

### The potential of endometriosis-specialist nursing roles

The cohort of nurses identified through this study who are interested, and in some cases highly motivated, to take on specialist endometriosis training could be key facilitators of better patient journeys through diagnosis and management of endometriosis in Aotearoa New Zealand. These nurses could be key in patient advocacy and education, particularly as those who are more interested in being an endometriosis-specialist nurse were also more interested in patient advocacy roles. This aligns with a UK study of endometriosis CNSs where 92% viewed that their role was being a patient advocate (Norton et al., 2020). Endometriosis patient experiences with medical practitioners are well characterised in Aotearoa New Zealand (Ellis and Wood, 2025) and overseas (Grundström et al., 2018), as having the capacity to either be destructive, through ignorance and disbelief or constructive, through acknowledgement of patients and facilitation of their journeys. In a participatory anthropological and semi-structured interviews study to determine nurse attitudes and actions in endometriosis care in hospital settings, Bach et al. (2016) compared endometriosis patient care in an endometriosis unit and an oncology unit. They identified an endometriosis patient could either be viewed as a worthy challenge and deserving of care or as a disturbance, depending on the exposure of the nurses to endometriosis patients regularly versus occasionally (and in comparison to their treatment of oncology patients) (Bach et al., 2016).

Nurses with an effective grounding in the fundamentals and specifics of endometriosis can empower patients, educate them about treatments and alternative pain control methods (e.g. diet or activity levels), facilitate diagnosis, offer emotional support, help patients cope with symptoms and complications, encourage open discussions, refer patients to local or online support groups and cultivate relationships with patients which allow for effective disease management and improvements in quality of life (Mao and Anastasi, 2010; Bloski and Pierson, 2008). Nurses are often under-utilised within healthcare systems and barred from being able to fully realise their potential to streamline patient care, practice patient-centred care and reduce patient presentation in acute care for management that could be conducted in a home or primary care setting (Flaubert et al., 2021). The interest of the majority of participants in this study in undertaking training to become an endometriosis-specialist nurse indicates that in Aotearoa New Zealand, there is a population of nurses that may be willing to take on the opportunity to upskill and take on more responsibility for endometriosis patients. This has also been identified amongst a cohort of Aotearoa New Zealand GPs as a key means to reduce the pressure on their services (Ellis et al., 2025). High-quality perioperative nursing care after laparoscopic treatment of endometriosis has already been highlighted as a key way to improve patient outcomes, particularly through reduction of the length of hospital stays (Dong et al., 2025; Liu et al., 2024). There are also preliminary indications that patients may welcome a widened role for nurses within their endometriosis care journey. In a sample of 844 German endometriosis patients, 91.8% felt it would be acceptable to have an ‘EndoNurse’ as a part of their care, indicating a very high perception of acceptability for incorporation of specialised nurses from a patient perspective too (Lukac et al., 2025).

In a UK survey of endometriosis CNSs, they explained that their roles encompassed and included being prescribers, ordering investigations, consenting for interventions, physical examinations, scanning, psychological support, making referrals, providing information, education about complementary therapies, auditing, conducting research and recruiting for research studies. Amongst the 38 endometriosis CNSs surveyed, 92% indicated that the activity that took up most of their time was administrative burdens. The median hours a week allocated to this role was only 13.5 hours (Norton et al., 2020).

### Strengths and limitations

Key strengths of this study include the high sample size with nurses that represent a range of age groups, ethnicities and nursing roles. Furthermore, this sample included nurses practising in all 16 regions of Aotearoa New Zealand, with the largest proportions from Auckland, Christchurch, and Wellington, which are also the regions with the highest proportions of population and practising nurses (Nursing Council of New Zealand | Te Kaunihera Tapuhi o Aotearoa, 2024). With regard to backgrounds in gynaecology or endometriosis, this nurse cohort is composed of almost a quarter of nurses whose workplace had an obstetrics and/or gynaecology specialisation, and 10% had done endometriosis-specific CME. The small sample sizes of the sub-groups disinterested in endometriosis-specialist nursing led to dichotomisation of groups for analysis which reduces the available statistical power and increases the chance for false negative findings. However, the key trends found with the dichotomised variables (reduced interest with age, and increased interest amongst those with frequent gynaecology consults) was also found during analysis of non-dichotomised data supporting the use of this analysis methodology (supplemental materials).

Only six (2.1%) of the nurses in the sample were male, meaning the study was underpowered to determine differences as a feature of sex. This represents an under-recruitment of male nurses for this study, as the population of nurses in Aotearoa New Zealand is 10% male (Nursing Council of New Zealand; Te Kaunihera Tapuhi o Aotearoa, 2024). This sample also over-represents primary care nurses. This may relate to the recruitment method predominantly targeting primary care clinics, as these were more likely to have a publicly available email address for the invitation to be sent to. The impact of the skew towards primary care nurses in the results is unable to be fully assessed in comparison to nurses from after-hours, hospital or community environments as this study did not have large enough sample sizes from these settings to assess differences. Consequently, these results should be viewed as a reflection of the views of predominantly primary care nurses. This study also largely over-represents New Zealand European nurses and somewhat over-represents New Zealand Māori nurses while under-representing Asian and Pasifika nurses (Nursing Council of New Zealand; Te Kaunihera Tapuhi o Aotearoa, 2024). This may relate to the predominance of primary care nurses in this study sample where proportions of New Zealand European and Māori nurses are higher (Nursing Council of New Zealand; Te Kaunihera Tapuhi o Aotearoa, 2024). Currently, the over-representation of New Zealand European nurses hinders the generalisability of these findings.

The collection of this data was done through a novel questionnaire which has not undergone formal validation, which limits overall validity. A strength to the questionnaire is that it was developed through adaptation of a previously published questionnaire used with GPs in an Aotearoa New Zealand context (Ellis et al., 2024), and further iterated through consultation with nurses and nursing groups about the relevance of content to their practice. Particular attention was paid to consultation with Māori nurses to ensure relevance to the context of their practices.

The final limitation that is important to note is that since participants in this study self-selected to participate in a survey about endometriosis, as in the comparable GP survey (Ellis et al., 2024), the results are likely biased towards nurses with greater interest in endometriosis care and patients. This means the study findings are likely skewed towards a higher level of endometriosis knowledge, awareness and interest. This indicates the proportion of nurses who perceived they had enough knowledge about endometriosis for their routine practice, 16.1%, may be an over-estimate. This is supported by the finding that nurses working in obstetrics and/or gynaecology focused clinics with likely more exposure to, and interest in endometriosis, felt they had enough knowledge.

### Implications

#### Nursing education

The findings from this cohort of nurses highlight key knowledge gaps, and a widespread sense that they do not know enough about endometriosis for their routine practice. It is expected that at least 10% of those presumed female at birth in Aotearoa New Zealand will have endometriosis (Manatū Hauora; Ministry of Health, 2022), whereas Australian data suggest the prevalence may be as high as 14% (Australian Institute of Health and Welfare, 2023). The commonness of this condition makes it highly likely that nurses from a wide range of practices will regularly have endometriosis patients in their care. To counteract the perception amongst these nurses that they do not know enough about endometriosis for their routine practice, widespread education programmes which address the key knowledge points on endometriosis best practice nurses need for their routine practice should be developed. These programmes should address both nurses already practising, and nurses in training. Furthermore, as best practice guidelines are developed to update or replace the *Diagnosis and Management of Endometriosis in New Zealand* guidelines, it would be beneficial to complement these guidelines with resources designed specifically for consumption by nurses.

#### Nursing practice and healthcare policy

This study highlights that there is a cohort of nurses who are very interested in training to be endometriosis-specialist nurses. Such an endometriosis-specialist nursing role would require training and accreditation, which should be designed in collaboration with the Nursing Council, the Royal Colleges of Obstetricians and Gynaecologists, and General Practitioners to ensure usefulness and effectiveness are maximised. Consultation with patients and patient organisations would also be valuable to ensure the role is fit for purpose to improve patient satisfaction and engagement with healthcare services. If developing an endometriosis-specialist nursing programme within the Aotearoa New Zealand context, it will be vital that there is sufficient funding for nurses to be able to focus on the endometriosis-specific element of their role to the degree that maximises the value for patient care.

#### Future research

The results of this exploratory study highlight a range of important areas for future research. Firstly, it would be valuable for an assessment of patient satisfaction and health outcomes when nurses are substituted for GPs for the acute and/or chronic care of endometriosis patients to highlight any key differences in this domain of healthcare. Secondly, the perception of acceptability of endometriosis-specialist nursing amongst Aotearoa New Zealand endometriosis patients should be determined. Thirdly, demographics that were under-represented within this cohort should be targeted for determination of any key differences, in particular, any unique limitations or barriers concerning endometriosis care and endometriosis-specialist nursing. This includes the recruitment of nurses operating within hospital or aftercare environments, and enhancing the participation of Asian and Pasifika nurses to ensure these perspectives can be captured in future research. Fourthly, research with nurses should compare and contrast these results with a randomly selected nurse cohort less likely to contain a bias towards an interest in addressing endometriosis care. This cohort could be recruited through an organisation such as the Nursing Council of Aotearoa New Zealand or utilise a cohort of student nurses. Future work should also look to further develop and validate this questionnaire so it can be readily utilised in a range of settings in and outside of the Aotearoa New Zealand context. Finally, work should be done to develop programmes and resources to empower nurses to feel that they have sufficient knowledge of endometriosis for their routine practice of caring for patients.

## Conclusions

This paper outlines the findings of the first study to assess Aotearoa New Zealand nurses’ awareness and understanding of endometriosis guidelines, perspectives of their role in this care, and interest in endometriosis-specialist nursing roles. Awareness of endometriosis guidelines and perception of having sufficient knowledge of endometriosis for their routine practice were both low amongst the nurses of this cohort. Furthermore, this study highlights there is a cohort of nurses in Aotearoa New Zealand who are interested in training to become endometriosis-specialist nurses. Development of any training programmes for endometriosis-specialist nursing roles should be done in a manner that will allow this training to be accredited while aligning with the key goals of interested nurses, practising healthcare practitioners and endometriosis patients. It will furthermore be vital to ensure there is sufficient funding and support for these specialised roles. Future research should look to determine differences in outcomes with nurse-led versus GP-led endometriosis care, further quantify the views and roles of nurses in endometriosis care in a range of settings in Aotearoa New Zealand and throughout the world, as well as the best ways to address the low uptake of nurses reading endometriosis guidelines and high prevalence of nurses perceiving they had insufficient knowledge of endometriosis for their routine practice.

**Key points for policy, practice and research**

- The New Zealand endometriosis guidelines have low readership amongst nurses, and nurses have a low self-perception of their endometriosis knowledge.
- Younger nurses had a greater emphasis on patient advocacy within their nursing roles for endometriosis patients, a generational difference with older nurses.
- About 72.9% of nurses indicated interest in endometriosis-specialist training. No such programme currently exists in Aotearoa New Zealand. There is international evidence to suggest that such a role could have a significantly positive influence on patient experiences and healthcare system function.
- Future research should seek to inform the collaborative design and implementation of a endometriosis-specialist nursing programme within the Aotearoa New Zealand context and assess consequent impacts on the care of endometriosis patients.

## Supplemental Material

sj-docx-1-jrn-10.1177_17449871261430232 – Supplemental material for Nursing care for patients with endometriosis in Aotearoa New Zealand: a survey study Supplemental material, sj-docx-1-jrn-10.1177_17449871261430232 for Nursing care for patients with endometriosis in Aotearoa New Zealand: a survey study by Katherine Ellis, Alina Meador, Anna Ponnampalam and Rachael Wood in Journal of Research in Nursing

sj-docx-2-jrn-10.1177_17449871261430232 – Supplemental material for Nursing care for patients with endometriosis in Aotearoa New Zealand: a survey study Supplemental material, sj-docx-2-jrn-10.1177_17449871261430232 for Nursing care for patients with endometriosis in Aotearoa New Zealand: a survey study by Katherine Ellis, Alina Meador, Anna Ponnampalam and Rachael Wood in Journal of Research in Nursing
