# Supplementary material for: Nursing care for patients with endometriosis in Aotearoa New Zealand: a survey study
Source: J Res Nurs. 2026 Apr 27:17449871261430232. Online ahead of print. doi: 10.1177/17449871261430232 (PMC13121231; doi:10.1177/17449871261430232)
Supplement: sj-docx-1-jrn-10.1177_17449871261430232 – Supplemental material for Nursing care for patients with endometriosis in Aotearoa New Zealand: a survey study [file sj-docx-1-jrn-10.1177_17449871261430232.docx]

**Methods S1.1. Non-dichotomised data analysis**

Interest in becoming an endometriosis-specialist nurse was measured on a seven-point Likert scale from very interested to very disinterested. To maintain the ordered nature of the seven-point Likert scale was transformed into a continuous variable with very interested responses were recoded as 7, interested as 6, somewhat interested as 5, neutral as 4, somewhat disinterested as 3, disinterested as 2, and very disinterested as 1. One-way ANOVA tests were conducted in Qualtrics StatsIQ to compare interest in specialist nursing against the age of respondents, and perception of personal endometriosis knowledge. T-tests were conducted to compare interest in specialist nursing with awareness and having read the endometriosis guidelines, completion of endometriosis-specific CME, frequency of gynaecology consults, whether their workplace had an obstetrics or gynaecology specialisation, and their level of experience as a nurse.

**Results S2.1. Interest in endometriosis-specialist nursing**

Those who had read the New Zealand endometriosis guidelines had a higher interest score in endometriosis-specialist scoring (mean 6.03 vs 5.13, t-test p = 0.000597), as did those that had done endometriosis-specific CME (mean 5.85 vs 5.17, t-test p = 0.0106), and those with frequent gynaecology consults (mean 5.54 vs 4.99, t-test p = 0.00584). Those who felt their knowledge about endometriosis was sufficient for their routine practice had a mean rating of 5.8/7.0 for interest in endometriosis-specialist nursing compared to 5.0 and 5.2 respectively (ANOVA p = 0.0167) for those who somewhat and did not feel their knowledge was sufficient. When compared by age (Table S1) those who were 20-29 gave a mean rating of 5.4, versus 5.6 for those 30-39, 5.6 for those 40-49, 5.1 for those 50-59 and 4.2 for those over the age of 60 (ANOVA p = 0.000426). The particular disinterest in the over 60s cohort differed significantly with only the 30-39 age group (p < 0.001) and the 40-49 age group (p < 0.001).

**Table S1: Differences in interest in endometriosis-specialist nursing by age group.**

|  | **20-29** | **30-39** | **40-49** | **50-59** | **60+** |
| --- | --- | --- | --- | --- | --- |
| **Very disinterested (1)** | 0.0% | 0.0% | 0.0% | 6.8% | 3.2% |
| **Disinterested (2)** | 5.3% | 0.0% | 2.5% | 0.0% | 16.1% |
| **Somewhat disinterested (3)** | 7.9% | 5.3% | 0.0% | 6.8% | 6.5% |
| **Neutral (4)** | 5.3% | 8.8% | 17.5% | 18.2% | 38.7% |
| **Somewhat interested (5)** | 28.9% | 29.8% | 22.5% | 27.3% | 19.4% |
| **Interested (6)** | 28.9% | 33.3% | 27.5% | 11.4% | 3.2% |
| **Very interested (7)** | 23.7% | 22.8% | 30.0% | 29.5% | 12.9% |
| **Mean rating** | 5.39 | 5.60 | 5.63 | 5.11 | 4.16 |
