# Supplementary material for: Nursing care for patients with endometriosis in Aotearoa New Zealand: a survey study
Source: J Res Nurs. 2026 Apr 27:17449871261430232. Online ahead of print. doi: 10.1177/17449871261430232 (PMC13121231; doi:10.1177/17449871261430232)
Supplement: sj-docx-2-jrn-10.1177_17449871261430232 – Supplemental material for Nursing care for patients with endometriosis in Aotearoa New Zealand: a survey study [file sj-docx-2-jrn-10.1177_17449871261430232.docx]

Appendix A: Survey Questions:

1. Demographics
   1. Do you identify as:

- Male
- Female
- Another Gender (Please Specify)
- Prefer Not to Say
  1. Are you:
- 18-19
- 20-29
- 30-39
- 40-49
- 50-59
- 60+
  1. Do you identify as (select all that apply):

Pākehā/New Zealand European

New Zealand Māori

Cook Islands Māori

Samoan

Tongan

Niuean

Tokelauan

Fijian

Other Pacific Peoples (Please Specify)

Chinese

Indian

Southeast Asian

Other Asian (Please Specify)

Middle Eastern

Latin American

North American

South American

African

European

Other (Please Specify)

- 1. Which region of New Zealand do you practice in:

Northland/Te Tai Tokerau

Auckland/Tāmaki-Makau-Rau

Bay of Plenty/Te Moana-a-Toi

Waikato

Taranaki

Gisborne/Te Tairāwhiti

Hawke’s Bay/Te Matau-a-Māui

Manawatū-Whanganui

Wellington/Te Whanga-nui-a-Tara

Tasman/Te Tai-o-Aorere

Nelson/Whakatū

Marlborough/Te Tau Ihu-o-te-Waka

West Coast/Te Tai Poutini

Canterbury/Waitaha

Otago/Ōtākou

Southland/Murihiku

- 1. Where have you trained (Please select all that apply):

New Zealand

Australia

North America

South America

Asia

Africa

UK & Europe

- 1. Is where you practice:
- Rural
- Semi-rural (Definition: “somewhat rural”)
- Urban
  1. Which best describes you:
     - Enrolled nurse
     - Registered nurse
     - Nurse practitioner
     - Senior nurse
     - Clinical nurse specialist
     - Nurse co-ordinator
     - Designated Māori Nurse
     - Designated Pasifika Nurse
     - Other (please specify)
  2. Is where you work primarily a:

Primary care clinic

After-hours or emergency clinic

Hospital

Community/NGO environment

Other (Please Specify)

- 1. Does where you work have an obstetrics and/or gynaecology specialisation?

Yes – obstetrics and gynaecology

Yes – obstetrics only

Yes – gynaecology only

No

- 1. After you finished your nursing qualification(s), did you do further gynaecological training:

No

Yes – Gynaecology internship

Yes – Gynaecology further education (post-graduate continuing education)

Yes – Gynaecology Diploma or similar

- 1. After you finished your nursing qualification, have you completed endometriosis-specific continuing medical education (CME):
- Yes
- No
  1. How frequently do you have gynaecology and/or women’s health consults or discussions with patients:

Never

Less than once per month

Several per month

Several per week

Every day

1. Endometriosis in your Practice
   1. Are you aware of the “Diagnosis and Management of Endometriosis in New Zealand” guidelines released by the Ministry of Health in 2020?

- Yes
- No
  1. Have you read the “Diagnosis and Management of Endometriosis in New Zealand” guidelines released by the Ministry of Health in 2020?
- Yes
- No
  1. (Only for 2.2. = Yes) Do you think the “Diagnosis and Management of Endometriosis in New Zealand” guidelines released by the Ministry of Health in 2020 are useful for your practice?
- Yes
- No
  1. Is there anything you wish to say about the “Diagnosis and Management of Endometriosis in New Zealand” guidelines released by the Ministry of Health in 2020? (Open-text)
  2. Do you feel you know enough about endometriosis for your routine practice?
- Yes
- Somewhat
- No
  1. Please rank the following for importance in your role as a nurse caring for endometriosis patients

Providing information about endometriosis as a disease

Providing information about endometriosis diagnosis

Providing information about endometriosis treatments

Providing information about endometriosis-related fertility implications

Providing emotional support for endometriosis patients

Advocating for endometriosis patients

Discussing potential lifestyle changes (e.g. diet or exercise changes)

Referring endometriosis patients to local or online support groups

- 1. Please rate the following symptoms out of 4 for diagnostic value for endometriosis.

1 = never has diagnostic value for endometriosis.

2 = sometimes has diagnostic value for endometriosis.

3 = often has diagnostic value for endometriosis.

4 = always has diagnostic value for endometriosis.

Dysmenorrhea (painful periods)

Deep dyspareunia (painful sex)

Infertility

Dysuria (painful urination)

Dyschezia (painful defecation)

Chronic pelvic pain (for a period of six months or more)

Painful rectal bleeding

Haematuria (blood in the urine)

- 1. What are the first-line treatments for symptomatic endometriosis? (Multi-choice)

Implant with etonogestrel (e.g. Levonorgestrel [Jadelle®])

Long-term treatment with NSAIDs (e.g. Ibuprofen)

GnRH (Gonadotrophin-releasing hormone) treatment (e.g. Goserelin [Zoladex®])

Progestin-only oral contraceptive pills (e.g. Desogestrel [Cerazette®])

Combined oral contraceptive pills (e.g. norethisterone/ethinylestradiol [Brevinor®])

Non-contraceptive progestins (e.g. medroxyprogesterone acetate [Provera®])

Long-term treatment with prescription-only pain relief (e.g. Codeine)

Surgery

IUCDS (Intra-uterine contraceptive device (e.g. Levonorgestrel [Mirena® or Jaydess®])

Progesterone injections (e.g. Medroxyprogesterone acetate [Depo-Provera®])

Other (Please specify)

- 1. Which of the following do you recommend to your patients that present with endometriosis symptoms? (Multi-choice)

Chronic pain clinic

Exercise

Acupuncture

Counselling/Talk-based therapy

Pregnancy

Diet changes

TENS (transcutaneous electrical nerve stimulation) machines

Supplements

Weight loss

Botox

Physiotherapy

Medicinal cannabis

Meditation

Other (Please specify)

- 1. How interested would you be in becoming an endometriosis-specialist nurse?

Very interested

Interested

Somewhat interested

Neutral

Somewhat disinterested

Disinterested

Very disinterested

- 1. (If somewhat-interested-very interested) What is the maximum amount of time you would be willing to dedicate to becoming an endometriosis specialist nurse? (Open-text)

1. Would you like to be contacted for a 20-30 minute discussion about your experiences with working with endometriosis patients? (Link to the information sheet)

- Yes – please follow the link to an interest form (which will not be connected with your answers to this survey) and we will send you a copy of information sheet and consent form for this follow-up study
- No
